# Supplementary material for: Structures of UBA6 explain its dual specificity for ubiquitin and FAT10
Source: Nat Commun. 2022 Aug 15;13:4789. doi: 10.1038/s41467-022-32040-6 (PMC9378703; doi:10.1038/s41467-022-32040-6)
Supplement: Supplementary file 1 — Supplementary Information [file 41467_2022_32040_MOESM1_ESM.pdf]

Supplementary Material

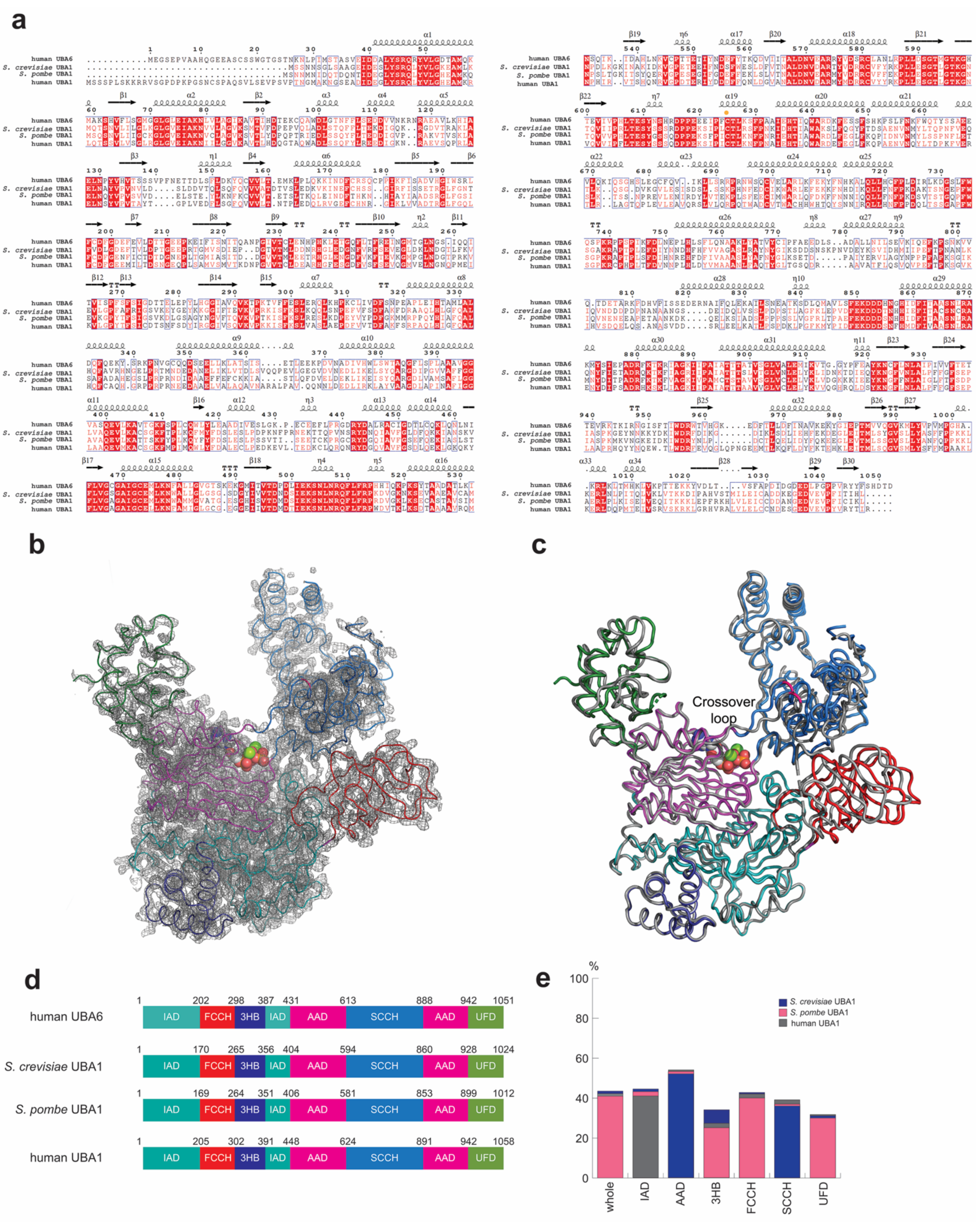

Figure S1. **Sequence and structural features of UBA6. a** Multiple sequence alignment of human UBA6 and the three structurally characterized UBA1 proteins (human UBA1, *S. cerevisiae* and *S. pombe* Uba1). Above the alignment, the secondary structure elements present in UBA6 are depicted

with helices as spirals (designated with α for α-helices and η for 3_{10}-helices) and β-strands as arrows. Sequences were aligned using Clustal Omega and the figure was generated with ESPript. Red shades indicate conserved residues, while red letters in white boxes indicate similar residues. The catalytically active cysteine (C625 of UBA6) is indicated by a pink dot. **b** SIGMAA weighted $2F_o$-F_c electron density map (gray) of the UBA6-ATP complex at a contour level of 1.0 rmsd. UBA6 is shown in loop representation with domains color coded as in Figure 1a-c and ATP in sphere representation. **c** Superimposition of the two protomers of the UBA6-ATP complex in loop representation with monomer A in domain coloring and monomer B in gray. **d** Domain architecture of UBA6 and UBA1 orthologs. **e** Overall and per domain sequence identity of UBA1 orthologs with respect to UBA6.

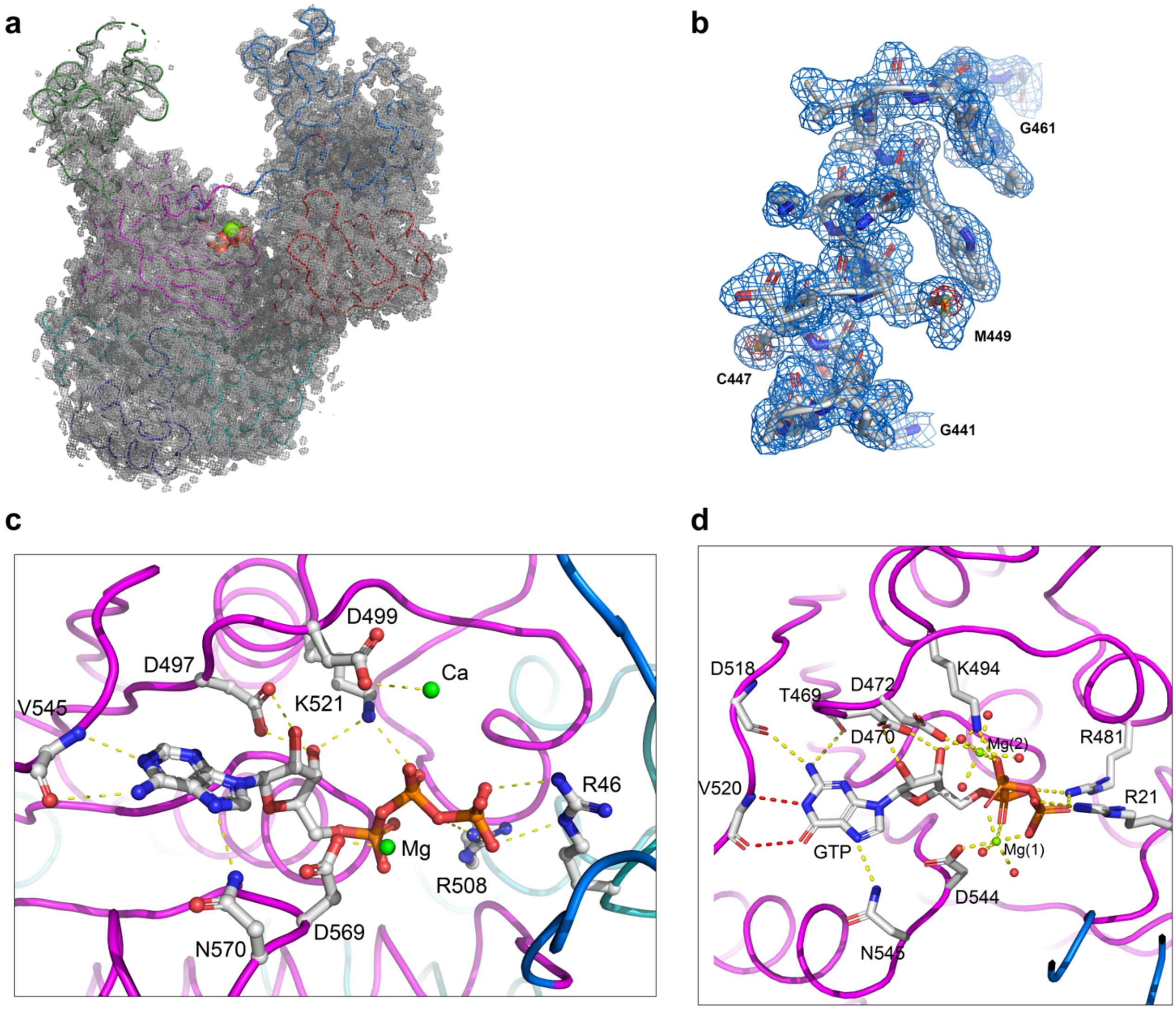

Figure S2. **Structural features of the Uba1-ATP complex.** **a** SIGMAA weighted $2F_o$-F_c electron density map (blue) of the Uba1-ATP complex at a contour level of 1.0 rmsd. Uba1 is shown in loop representation with domains color coded as in Figure 1a-c for UBA6. **b** SIGMAA weighted $2F_o$-F_c electron density map of residues G441-G461 in the Uba1-ATP complex at contour levels of 1.3 rmsd (blue) and 6 rmsd (red), with the latter revealing the positions of the two S-atoms present in this part of the model. The first and last residue together with the S-containing residues are labeled. **c** Coordination of the nucleotide cofactor in the UBA6-ATP structure with direct interactions highlighted as dashed yellow lines. The Mg and Ca ion are indicated but due to the more limited resolution, the water molecules coordinating the metals have been omitted. **d** Model of the Uba1-GTP complex. Additional hydrogen bonds involving the exocyclic amino group of guanine and the main chain oxygen atoms of T469 and D518 are shown in yellow, while repulsive interactions with the main chain of V520 are shown in red.

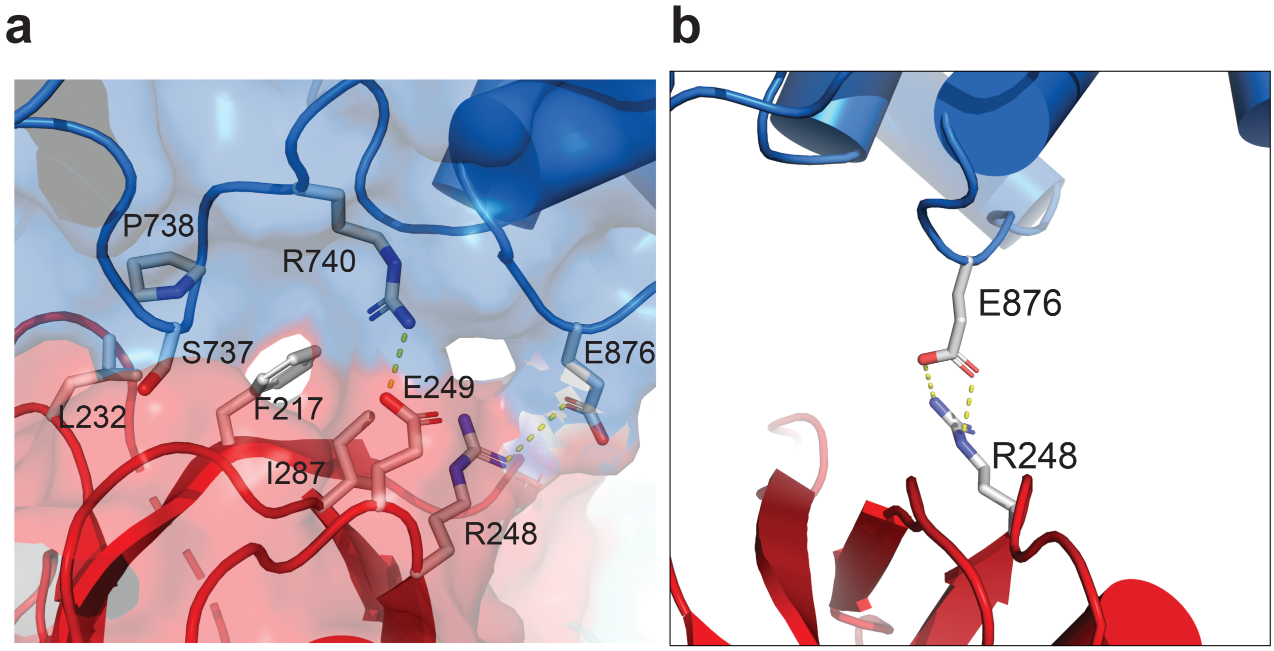

c

```
                                 FCCH
human UBA6         215 EIFISNITQANPGIVTCLENHPHKLETGQFLTFRE 249
S. cerevisiae UBA1 183 TGMVSDIEP--DGTVTMLDDNRHGLEDGNFVRFSE 215
S. pombe UBA1      182 TGMIASITD--DGVVTMLEETRHGLENGDFVKFTE 214
human UBA1         218 SAMVSMVTKDNPGVVTCLDEARHGFESGDFVSFSE 252
conservation             ::: :     * ** *::  * :* *:*: * *

                           SCCH
human UBA6         737 SPKRP 741      874 SIEPA 878
S. cerevisiae UBA1 710 GAKRA 714      846 FIETA 850
S. pombe UBA1      704 GPKRA 708      835 DITPA 840
human UBA1         744 GPKRC 748      874 DIPSA 879
conservation           . **                *  *
```

Figure S3. **Stabilization of the FCCH in the outward conformation. a** Interface between the FCCH and SCCH domains of the UBA6-ATP complex (ribbon representation with transparent surface) in monomer B. **b** Ion pair stabilizing the FCCH and SCCH domains in the UBA6-ATP complex in monomer A. **c** Partial sequence alignment of UBA6 and UBA1 orthologs showing the conservation of ionic interactions between the FCCH and SCCH domains.

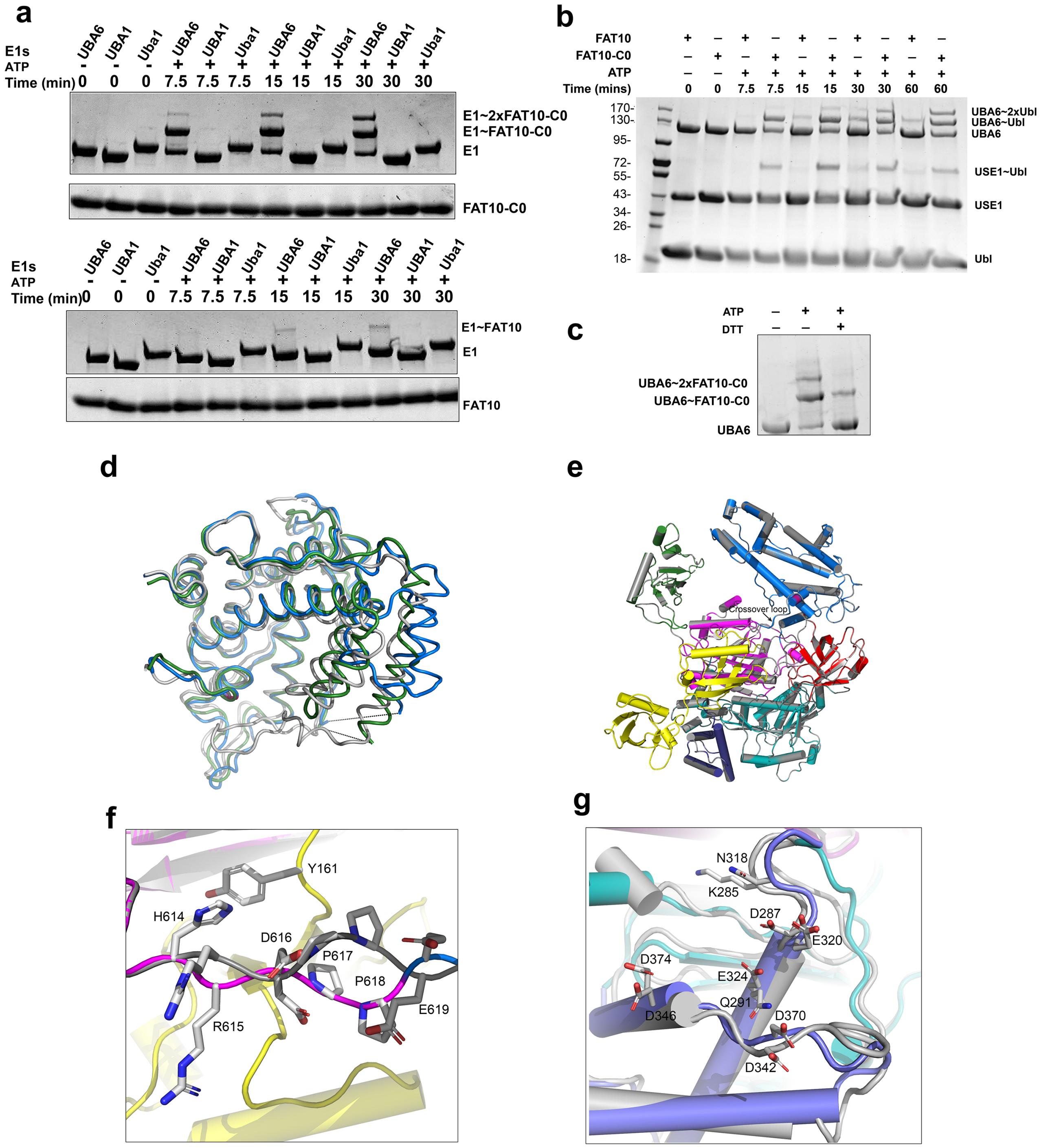

Figure S4 **Activity assays and structural analysis of the UBA6-FAT10 complex.** **a** UBA6, human UBA1 and *S. cerevisiae* Uba1 activity assay comparing the activation of FAT10-C0 (top) and the FAT10 wild-type (bottom). **b** UBA6-USE1 transthioesterification assay comparing the activation of the FAT10 wild-type and FAT10-C0. **c** UBA6 activity assay run for 40 min in the absence and presence of DTT showing the disappearance of the UBA6+2xC0-FAT10 band upon DTT treatment. The assays in a-c were repeated 3 times with similar results, source data are provided as a Source Data file. **d** Superimposition of the SCCH domains of UBA6 from the UBA6$_{chim}$-FAT10 complex

(marine blue), from the UBA1-ATP complex (green) and the Uba1-ATP complex (gray). **e** Superimposition of the UBA6-FAT10 complex (UBA6 is color coded according to its domains and FAT10 in yellow) with the apo-UBA6 monomer (gray) present in the P1 unit cell. **f** Superimposition of the UBA6-FAT10 complex highlighting the N-terminal region of the crossover loop with side chains undergoing conformational changes upon FAT10 binding, together with Y161 in the C-terminal tail of FAT10. **g** Comparison of the region in the 3HB of UBA6 (color coded according to its domains) interacting with the NTD of FAT10 and Uba1 (gray). Residues of UBA6 interacting with FAT10 and their counterparts in Uba1 are highlighted.

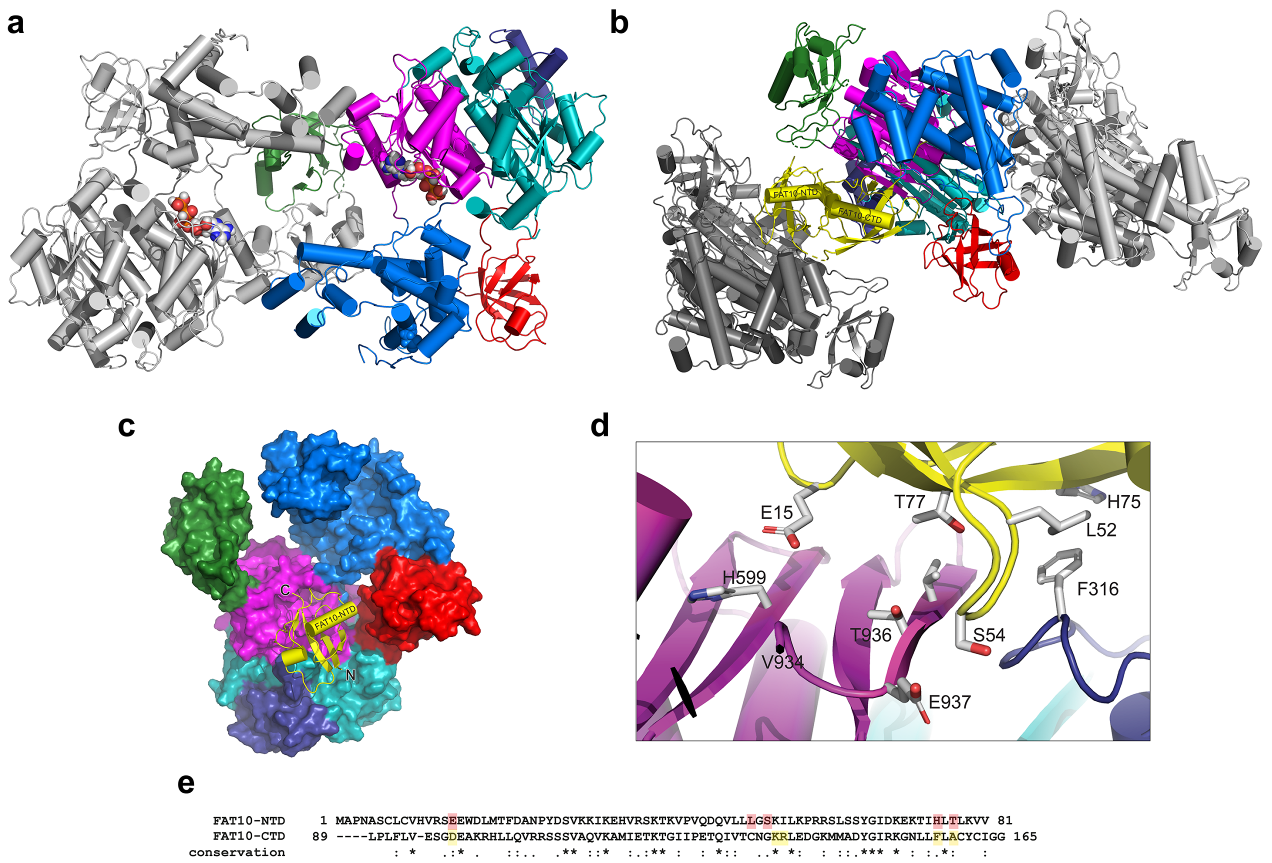

Figure S5. **Analysis of the UBA6-FAT10 complex. a** Arrangement of the two UBA6-ATP complexes in the asymmetric unit of the C2 cell. One complex is color coded according to its domains and the other is in light gray with ATP in space filling representation. The view is along the non-crystallographic twofold axis of symmetry. **b** Arrangement of the two UAB6$_{chim}$ molecules (one color coded according to its domains and the other in light gray) and FAT10 (yellow) in the P1 unit cell. The two UBA6 $_{chim}$ molecules are related by a non-crystallographic twofold axis of symmetry coinciding with the viewing direction. A symmetry-related UBA6$_{chim}$ molecule is shown in dark gray to illustrate how FAT10 is simultaneously interacting with the UBA6$_{chim}$ molecule shown in color and the symmetry-related UBA6 (dark gray). **c** Overall view of the interaction between the symmetry related UBA6$_{chim}$ molecule from **b** but now shown in surface representation and color coded according to its domain architecture illustrating its interaction with the NTD of FAT10. **d** Detailed interactions between the NTD of FAT10 and the symmetry-related UBA6$_{chim}$ molecule. **e** Sequence alignment of the NTD and CTD of FAT10 highlighting residues interacting with UBA6.

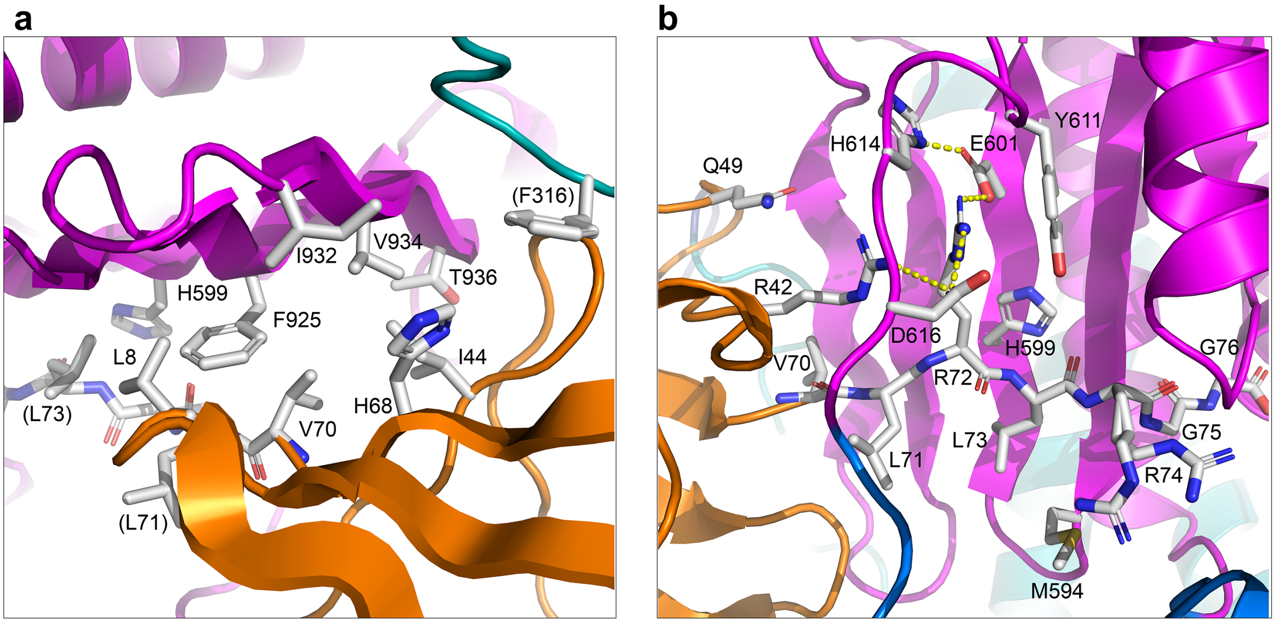

Figure S6**. Features of the UBA6-ubiquitin model. a** Hydrophobic interface in the model of the UBA6-ubiquitin complex. UBA6 is color coded according to is domains while ubiquitin is in orange. Residues contributing to the hydrophobic interface are shown with their side chains. To minimize steric clashes the side chain of L8 was modeled with a different rotamer. Residue labels in parentheses indicate side chains which are not involved in hydrophobic interactions but are included as reference points. The orientation resembles that of the UBA6-FAT10 complex shown in Figure 4d. **b** Interactions between the C-terminal tail of ubiquitin and UBA6 in the UBA6-ubiquitin model. H614 and D616 were modeled with different rotamers to allow binding of R72. R74 is represented by two alternate conformations as observed in the Uba1-ubiquitin complex.

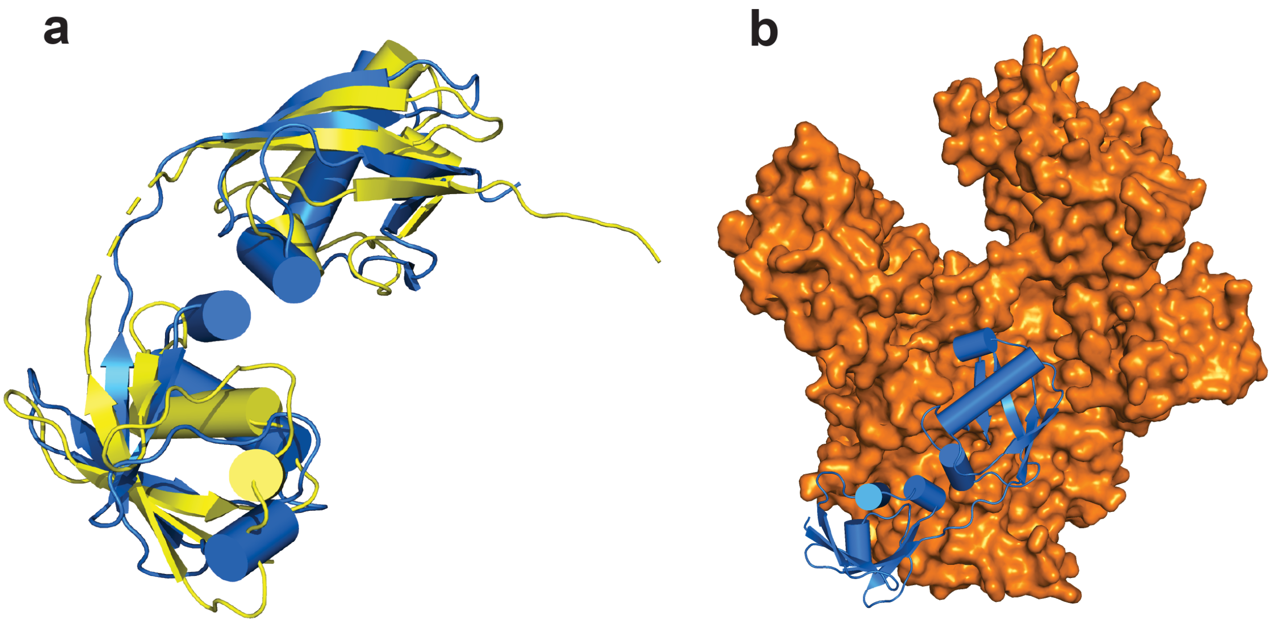

Figure S7 **Model of the UBA7-ISG15 complex. a** Superimposition of the FAT10 structure (yellow) as observed in the UBA6$_{chim}$-FAT10 complex with the crystal structure of ISG15 (PDB entry 1z2m) (blue) based on both domains. **b** Model of the UBA7-ISG15 complex. UBA7 is shown in surface representation (orange) and ISG15 in ribbon representation (blue). Based on the UBA6$_{chim}$-FAT10 complex UBA6 was replaced with the Alphafold model of UBA7 and the CTD of ISG15 was superimposed with its counterpart in FAT10.

Supplementary Table 1

Nr.	Original sequence	Mutation	Sequence
**1**	pGEX-del34UBA6	F316A fw	tgccttattgtggatgctagcaaccctgaggcacctttag
		F316A rv	cctcagggttgctagcatccacaataaggcactttgg
**2**	pGEX-del34UBA6	V934A fw	ccattccaattgtagcatttacagagacaactgaag
		V934A rv	ttgtctctgtaaatgctacaattggaatggctaag
**3**	pGEX-del34UBA6	E320K fw	gcaaccctaaggcacctttagagattc
		E320K rv	gaatctctaaaggtgccttagggttgctaaaatcc
**4**	pGEX-del34UBA6	E324R fw	cctgaggcacctttacggattcacacagctatgc
		E324R rv	gctgtgtgaatccgtaaaggtgcctcagg
**5**	pGEX-del34UBA6	D370R fw	ggaagagaagcctcgtgtaaatgctgacattgtg
		D370R rv	cacaatgtcagcatttacacgaggcttctcttcc
**6**	pGEX-del34UBA6	D616A fw	caatagtcatcgggctcccccagaagagg
		D616A rv	cctcttctgggggagcccgatgactattg
**7**	pGEX-del34UBA6	E601Q fw	aagggacacactcaagttattgtaccgcatttg
		E601Q rv	gtacaataacttgagtgtgtcccttagtgcccattg
**8**	pET28a-ScUba1	D472A fw	gttggacttttcgatcgaagcgttatccgtaacaacgat
		D472A rv	atcgttgttacggataacgcttcgatcgaaaagtccaac
**9**	pET28a-ScUba1	D472E fw	gacttttcgatcgactcgttatccgtaacaacgatgtaaccg
		D472E rv	cggttacatcgttgttacggataacgagtcgatcgaaaagtc
**10**	pET28a-ScUba1	D544A fw	tcttgcgtcgacattggctagagcgttggtgac
		D544A rv	gtcaccaacgctctagccaatgtcgacgcaaga
**11**	pET28a-ScUba1	D544E fw	gttcttgcgtcgacattctctagagcgttggtgac
		D544E rv	gtcaccaacgctctagagaatgtcgacgcaagaac
**12**	pET28a-ScUba1	K494E fw	cctctgccgctacttcagactcgttctttccgacatccttt
		K494E rv	aaaggatgtcggaaagaacgagtctgaagtagcggcagagg
**13**	pET28a-ScUba1	R21A fw	cccaacacataaagttgagcagaataaagactttcatcgatttctcc
		R21A rv	ggagaaatcgatgaaagtctttattctgctcaactttatgtgttggg
**14**	pET28a-ScUba1	R481A fw	ctttggtctaaacaagaactgagcgtttaagttggacttttcgatc
		R481A rv	gatcgaaaagtccaacttaaacgctcagttcttgtttagaccaaag
**15**	pET28a-ScUba1	C600A fw	ttgggaaagaacgtagggtagccaatgggatagacttttctg
		C600A rv	cagaaaagtctatcccattggctaccctacgttctttcccaa
**16**	pGEX-del34UBA6	C625A fw	gaggaaataccatttgctactctaaaatcctttcc
		C625A rv	attttagagtagcaaatggtatttcctcttctgg
**17**	pGEX-del34UBA6	UBA6chim fw1	aagaggaaataccaatctgtaccctgaagaac
		UBA6chim rv1	tcttcagggtacagattggtatttcctcttc
	huUba1-pET23b	UBA6chim fw2	aagctgattgcagggaaaattatacctgctatag
		UBA6chim rv2	agcaggtataattttccctgcaatcagcttgc
